# Advancements in the Analysis of the *Arabidopsis* Plasma Membrane Proteome

**DOI:** 10.3389/fpls.2013.00086

**Published:** 2013-04-11

**Authors:** Koste A. Yadeta, J. Mitch Elmore, Gitta Coaker

**Affiliations:** ^1^Department of Plant Pathology, University of California DavisDavis, CA, USA

**Keywords:** plasma membrane, proteomics, mass spectrometry, membrane proteins, *Arabidopsis*

## Abstract

The plasma membrane (PM) regulates diverse processes essential to plant growth, development, and survival in an ever-changing environment. In addition to maintaining normal cellular homeostasis and plant nutrient status, PM proteins perceive and respond to a myriad of environmental cues. Here we review recent advances in the analysis of the plant PM proteome with a focus on the model plant *Arabidopsis thaliana*. Due to membrane heterogeneity, hydrophobicity, and low relative abundance, analysis of the PM proteome has been a special challenge. Various experimental techniques to enrich PM proteins and different protein and peptide separation strategies have facilitated the identification of thousands of integral and membrane-associated proteins. Numerous classes of proteins are present at the PM with diverse biological functions. PM microdomains have attracted much attention. However, it still remains a challenge to characterize these cell membrane compartments. Dynamic changes in the PM proteome in response to different biotic and abiotic stimuli are highlighted. Future prospects for PM proteomics research are also discussed.

## Introduction

The plasma membrane (PM) is the cellular interface that regulates the exchange of molecules and information between cells and their environment. The PM is involved in a range of plant physiological processes including growth and development, ion and metabolite transport, perception of environmental changes, and disease resistance (Marmagne et al., 2004; Mongrand et al., 2010). At the cellular level, PM proteins maintain the electrochemical gradients required for membrane transport and play a critical role in osmoregulation of the cell (Schulz, 2011). In addition, the PM plays an essential role in sensing and responding to biotic and abiotic stresses. PM transporters control the distribution and movement of plant hormones and thus mediate short- and long-distance signaling processes (Kerr et al., 2011). Various plant hormone receptors for auxin, brassinosteroids (BR), and abscisic acid are also localized to the PM (Wang et al., 2001; Pandey et al., 2009; Robert et al., 2010). In addition, many plant innate immune receptors and defense response regulators are integrally or peripherally associated with the PM (Dodds and Rathjen, 2010; Monaghan and Zipfel, 2012), highlighting the importance of the PM in regulating numerous aspects of plant growth, development, and adaptation to changing environments.

The PM is composed of a lipid bilayer and associated proteins. Plant cell membrane lipids consist primarily of glycerophospholipids, sphingolipids, and sterols (Furt et al., 2011; Cacas et al., 2012). Membrane proteins can be directly embedded within the lipid bilayer or undergo lipid modification which impacts their localization and membrane association. For many years, the biological membrane was considered as a dynamic two-dimensional fluid composed of homogenously distributed lipids and proteins (Singer and Nicolson, 1972). However, now it is clear that distinct membrane microdomains of various sizes and mobilities exist in eukaryotic cells (Kusumi et al., 2012). In plants, PM microdomains have been implicated in various cellular processes including cell wall attachment, protein sorting and trafficking, signal transduction, and plant–microbe interactions (Mongrand et al., 2010; Simon-Plas et al., 2011; Urbanus and Ott, 2012). In addition to membrane compartmentalization, post-translational protein modifications (PTMs) of PM proteins impacts their activity and signaling capabilities. Currently, over 300 different PTMs have been reported, with protein phosphorylation being the most intensively studied (Zhao and Jensen, 2009; Kline-Jonakin et al., 2011). PTMs can modulate protein activity through changes in protein conformation, localization, stability, and protein–protein interactions. Global surveys and functional analysis of protein PTMs during signaling events are now possible through advancements in proteomic approaches (Zhao and Jensen, 2009).

In eukaryotes, roughly 30% of the genome encodes membrane proteins (Tan et al., 2008). However, in many studies PM proteins are often underrepresented due to their physiochemical heterogeneity, hydrophobicity, and low abundance (Marmagne et al., 2007). Several technological advances have been developed that overcome some of the challenges afflicting PM proteomics analyses. Various label-based and label-free methods exist for the quantification of peptides, proteins, and PTMs in plant tissue extracts (as reviewed in Schulze and Usadel, 2010; Kline-Jonakin et al., 2011; Kota and Goshe, 2011). Here we briefly summarize recent advancements in PM proteomics with a focus on the model plant *Arabidopsis thaliana*.

## Evolution of Plasma Membrane Proteomic Strategies: Enrich, Solubilize, and Analyze

**Figure 1:**
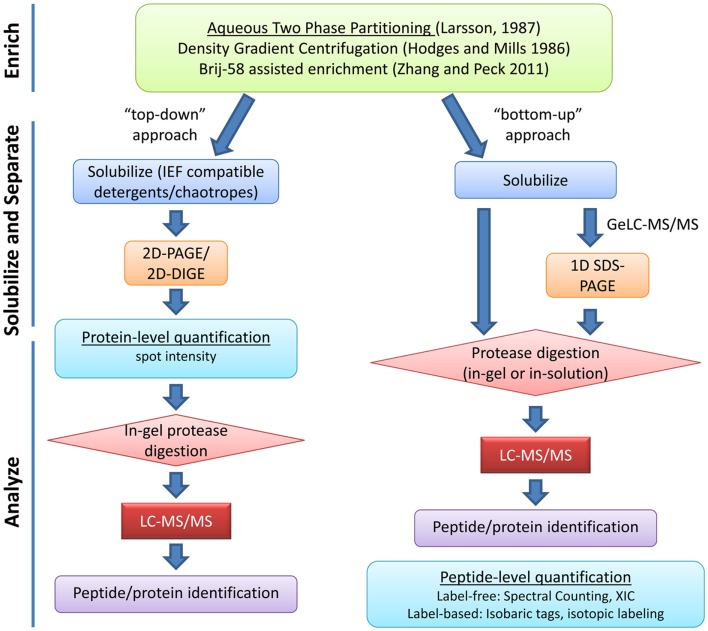
**Overview of typical plasma membrane proteomics experiments**. Enrichment of PM proteins is usually achieved through aqueous two-phase partitioning. Strategies for solubilization and separation of PM proteins are chosen based on downstream applications. “Top-down” approaches use protein-level quantification while “bottom-up” approaches use peptide-level quantification. Various strategies for label-based and label-free peptide quantification exist and will influence experimental design (reviewed in Schulze and Usadel, 2010; Kota and Goshe, 2011). XIC, extracted ion chromatogram.

Due to the unique roles of the PM in cellular function, identification, and functional characterization of the plant PM proteome has been critical for understanding of how plants grow, develop, and respond to their environment. The low relative abundance of PM proteins in whole tissue extracts has necessitated the development of various strategies to enrich for proteins specific to the cell membrane before proteomic analysis. Even after isolation of PM fractions, due to the complexity of protein species and the large dynamic range of protein abundance, it is necessary to employ various protein and/or peptide separation techniques to achieve a comprehensive survey of the PM proteome (Figure 1).

Various techniques have been used for PM separation and enrichment from total microsomal fractions including: density gradient centrifugation, free-flow electrophoresis, and phase polymer systems (Larsson et al., 1987; Dunkley et al., 2006; Santoni, 2007). Aqueous two-phase partitioning is one of the most common and effective techniques for enrichment of PM vesicles to high purity (Larsson et al., 1987). Above a critical concentration, the polymers polyethylene glycol (PEG) and dextran will form distinct phases when mixed in aqueous solution. Biomembranes associate with one phase or the other based on the membrane surface charge (Larsson et al., 1987). PM vesicles preferentially partition in the PEG-enriched upper phase while other cellular membranes associate with the dextran-enriched lower phase. Repeated cycles of phase partitioning can result in highly enriched PM fractions estimated to be over 90% pure based on enzyme marker assays (Larsson et al., 1987; Palmgren et al., 1990). Recently, a relatively simple approach for enrichment of PM proteins has been described (Zhang and Peck, 2011). Although the resulting PM fractions are not as pure as those derived from two-phase partitioning, this method is rapid and requires less sample handling, making it advantageous in situations where many samples must be processed at the same time (Zhang and Peck, 2011).

Because subcellular compartments isolated using biochemical approaches are never 100% pure, it is of interest to reduce and then evaluate contaminating organelles/proteins when isolating PMs. Testing PM enrichment and purity relative to total microsomal fractions usually involves enzymatic assays of the PM H+-ATPase or various immunological markers (Larsson et al., 1987; Marmagne et al., 2007). Because PM vesicles isolated by two-phase partitioning are predominantly apoplastic-side out, the non-ionic detergent Brij-58 is commonly used to invert PM vesicles inside-out and release organelles and/or cytosolic proteins that are trapped within the vesicles during tissue disruption (Palmgren et al., 1990; Johansson et al., 1995; Zhang and Peck, 2011). If the goal is to analyze primarily integral membrane proteins (IMPs), high pH and/or high salt treatments of inverted vesicles can be used to remove peripheral and loosely associated cytosolic proteins from the membrane (Santoni et al., 1999; Santoni, 2007; Marmagne et al., 2007). These treatments have facilitated the identification of a larger number of hydrophobic proteins after PM enrichment (Marmagne et al., 2007).

In order to evaluate contaminating proteins in PM preparations, quantitative isotopic labeling has been used for fractions enriched by density gradient centrifugation (Dunkley et al., 2006) and aqueous two-phase partitioning (Nelson et al., 2006). By comparing the degree of enrichment of known PM protein markers (e.g., PM H^+^-ATPase) in a given fraction relative to other subcellular markers, it was possible to confidently assign PM localization to a number of previously unknown PM proteins in *Arabidopsis* (Dunkley et al., 2006; Nelson et al., 2006). Using this approach, it was estimated that over 25% of proteins identified in PM-enriched fractions could be considered biological contaminants (Nelson et al., 2006). However, often in functional proteomics investigations, the goal is not to achieve absolutely pure PM fractions, but to enrich PM proteins in order to study the behavior of the PM during a physiological process or under stress conditions (Zhang and Peck, 2011). Thus it is less important to unequivocally assign a subcellular location to a protein, than to reproducibly identify and quantify its behavior under a specific condition (Elmore et al., 2012). Nevertheless, several excellent resources are available for the analysis and validation of proteins identified from a PM proteomics experiment (Table 1).

**Table 1:** **Web-based resources for protein analyses and validation**.

Database	Description	Website	Reference
**SUBCELLULAR LOCATION/TRANSMEMBRANE (TM) PREDICTION**			
ARAMEMNON	Consensus prediction of TM domains, lipid modification, signal peptides, and subcellular location; contains link to all prediction programs	http://aramemnon.botan ik.uni-koeln.de/	Schwacke et al. (2003)
SUBA3	Combines subcellular prediction programs with experimental data (GFP localization, MS/MS, etc)	http://suba.plantenergy.uwa.edu.au/	Heazlewood et al. (2007)
ExPASy	Collection of multiple tools for the prediction of post-translational modifications and protein localization	http://www.expasy.org/p roteomics/post-translational_modification	Artimo et al. (2012)
**PROTEIN PHOSPHORYLATION**			
PhosPhAt	Aggregation of phosphorylation sites identified by mass spectrometry collected from over 20 studies in *Arabidopsis*. Can visualize raw MS/MS spectrum data	http://phosphat.mpimp-golm.mpg.de/	Durek et al. (2010)
P3DB	Plant protein phosphorylation site database. Can visualize raw MS/MS spectrum data	http://www.p3db.org/	Yao et al. (2012)
PlantsP	Functional genomics database focusing on protein kinases and phosphatases	http://plantsp.genomics.purdue.edu/index.html	Tchieu et al. (2003)
**DATA AGGREGATION/GENERAL PROTEOMICS**			
MaSCP gator	Constantly updated data aggregation portal that retrieves proteomics information from several actively curated databases	http://gator.masc-proteomics.org/	Joshi et al. (2011)
pep2pro	Searchable mass spectrum library of experimentally identified peptides in MS/MS studies	http://fgcz-pep2pro.uzh.ch/	Hirsch-Hoffmann et al. (2012)
MetNet	Systems biology tool for combined analysis of protein, gene expression, and metabolite profiling data	http://www.metnetonline.org/	Sucaet et al. (2012)
**PROTEIN–PROTEIN INTERACTIONS**			
MIND	Membrane protein–protein interaction dataset using yeast split-ubiquitin system	http://www.associomics.org/Associomics/MIND.html	Lalonde et al. (2010)
ANAP	Aggregates gene and protein interaction data from diverse sources. Web portal for cytoscape visualization	http://gmdd.shgmo.org/Computational-Biology/ANAP/ANAP_V1.1/	Wang et al. (2012)

After PM isolation, proteomics analyses typically involve both gel-based and gel-free methods for separation of proteins or peptides prior to identification by mass spectrometry (MS). Early efforts in *Arabidopsis* utilized two-dimensional gel electrophoresis (2DGE) but later it became clear that 2DGE was not an ideal technique for separation of membrane proteins (Santoni et al., 1998, 2000; Prime et al., 2000). Most hydrophobic proteins have limited solubility in buffers required for the first dimension isoelectric focusing (IEF) step of 2DGE (Wilkins et al., 1998; Santoni et al., 2000). The low abundance, hydrophobicity, generally large molecular weight, and generally alkaline nature of PM proteins have all led to the poor performance of 2DGE in PM proteomics (Santoni et al., 2000; Gilmore and Washburn, 2010). Nevertheless, various chaotropes and detergents have been used with improvements in solubility and resolution of some membrane proteins in 2DGE and this “top-down” approach has been used with success to study hormone signaling at the PM (Santoni et al., 1999; Luche et al., 2003; Tang et al., 2008a) (Figure 1).

For most researchers, liquid chromatography-tandem MS (LC-MS/MS) “bottom-up” shotgun proteomics has emerged as the method of choice for large-scale identification and quantification of proteins, especially membrane proteins (Figure 1). PM protein samples are first solubilized, digested with a protease to cleave polypeptide chains into shorter peptide fragments, and then these fragments are separated by LC prior to ionization and MS/MS analysis. Various PM protein solubilization strategies prior to in-solution or in-gel digestion have been used to increase coverage of PM proteins in LC-MS/MS analysis (Marmagne et al., 2004; Mitra et al., 2007).

After digestion, peptides can be separated in one or more dimensions, typically involving reverse phase (RP) and/or strong cation exchange (SCX) chromatography for increased resolution and improved detection of low abundance peptides (Washburn et al., 2001; Fournier et al., 2007; Gilmore and Washburn, 2010). The use of different proteases with varying cleavage specificities has also increased the representation of membrane proteins in MS/MS datasets (Wu et al., 2003; Fischer and Poetsch, 2006). Recent reviews focus on advances in protein and peptide separation strategies for MS-based membrane proteomics (Fournier et al., 2007; Komatsu, 2008; Gilmore and Washburn, 2010).

One method gaining popularity is Gel-enhanced LC-MS/MS (GeLC-MS/MS), where extracted proteins are first subjected to one dimensional SDS-PAGE to separate by size and then regions of the gel lane are excised, digested, and subjected to LC-MS/MS separately (Alexandersson et al., 2004; Marmagne et al., 2007; Gilmore and Washburn, 2010). GeLC-MS/MS has been shown to outperform other separation techniques in terms of reproducibility and total number of protein identifications (Fang et al., 2010; Piersma et al., 2010). Another advantage of the GeLC-MS/MS approach is that PM fractions can be efficiently solubilized in strong detergents and/or chaotropes prior to SDS-PAGE, then digested in-gel to yield peptides suitable for MS/MS analysis.

## Major Classes of Proteins in the *Arabidopsis* PM and Their Biological Functions

The PM consists of structurally and functionally diverse proteins. The composition of the PM proteome varies with plant cell-type, developmental stage, and environmental conditions (Alexandersson et al., 2004). PM proteins can be classified into three main categories depending on the type of membrane association: IMPs, peripheral membrane proteins (PMPs), and glycosylphosphatidylinositol (GPI)-anchored membrane proteins. Many resources exist for the prediction of PM localization, transmembrane (TM) domains, lipid-based modifications, and GPI-anchors in proteins identified from PM fractions (Schwacke et al., 2003; Heazlewood et al., 2007) (Table 1).

### Integral membrane proteins

Integral membrane proteins are composed of one or more hydrophobic TM domains that span the lipid bilayer of the membrane. The majority of IMPs span the lipid bilayer with an α-helical structure, although some IMP domains exhibit β-barrel structure (Marmagne et al., 2007; Tan et al., 2008). Many IMPs contain a N-terminal signal peptide for secretion and membrane targeting through the ER and Golgi. Most active and passive membrane transport processes are controlled by a variety of IMP pumps, channels, and carriers (Schulz, 2011). One of the most abundant proteins in the plant PM, the PMH^+^-ATPase, is the primary proton pump responsible for the establishment of the electrochemical gradient across the membrane that drives secondary transport processes. Other highly abundant PM proteins include the PM intrinsic protein (PIP) or aquaporin family which function mainly as water channels but can transport other small molecules (Schulz, 2011). Various other ion, hormone, and nutrient transporters exist at the PM as IMPs. ATP-binding cassette (ABC) transporters use ATP hydrolysis to drive the efflux or influx of a variety of substances including auxin, ABA, heavy metals, and antimicrobial compounds (Schulz, 2011) (Figure 3).

In addition to membrane transport activities, other IMPs are involved in the perception of extracellular signals and activation of downstream responses. One of the largest classes of signaling proteins in plants is the Receptor-like kinase (RLK) family, whose members can be relatively abundant on the PM (Santoni et al., 2003; Alexandersson et al., 2004; Marmagne et al., 2004). Characterized *Arabidopsis* RLKs function in a variety of processes including cell division and differentiation, hormone perception, meristem maintenance, pathogen recognition, and cell death control (De Smet et al., 2009; Monaghan and Zipfel, 2012).

### Peripheral membrane proteins

Peripheral membrane proteins lack a membrane spanning domain but are membrane-associated either through covalent lipid modifications or non-covalent protein–protein interactions (Marmagne et al., 2007; Tan et al., 2008). Lipid modifications such as N-myristoylation, S-palmitoylation, or prenylation are common in PMPs and these modifications can control protein localization, sorting, and function (Testerink and Munnik, 2011). Proteins involved in vesicular membrane trafficking such as Rho of plants (ROPs) and Soluble N-ethylmaleimide sensitive factor attachment protein receptors (SNAREs) are commonly targeted to the PM via lipid modification (Sanderfoot et al., 2000; Testerink and Munnik, 2011). In addition to these lipid PTMs, many proteins associate with the PM via protein–protein interactions. These types of PMPs are often involved in signaling events by relaying messages from the PM to the rest of the cell.

### GPI-anchored membrane proteins

Glycosylphosphatidylinositol-anchored membrane proteins are post-translationally modified to carry a C-terminal GPI-anchor that mediates their association with the membrane. The GPI-anchor is synthesized in endoplasmic reticulum and subsequently attached to a protein, which is transported to PM via the Golgi (Elortza et al., 2003; Fujita and Kinoshita, 2012). Unlike most PMPs, which localize to the cytosolic side of the PM, GPI-anchored membrane proteins are mostly found attached to the outer surface of the PM. Many enzymes associated with cell wall processes (e.g., β-1,3-glucanases, pectinesterases, and polygalacturonases) are among the GPI-anchored proteins identified in *Arabidopsis* PM proteome (Borner et al., 2003; Elortza et al., 2003). Accordingly, GPI-anchored proteins are implicated in biological processes such as directional cell expansion, cellulose deposition, cell wall attachment and remodeling, and plant immunity (Elortza et al., 2006; Fujita and Kinoshita, 2012). GPI-anchored proteins are often found enriched in membrane microdomain preparations (discussed below), suggesting that cell wall maintenance hubs are compartmentalized within the membrane (Kierszniowska et al., 2009).

### The dynamic plasma membrane

Proteins are constantly associating and disassociating from the membrane during endocytic, secretion, and signaling events. Enzyme activity, signal transduction, and transport regulation are all influenced by post-translational modifications that can affect the function and/or localization of proteins at or within the PM without changing their overall abundance. Even within a cell, polarized distribution of proteins involved in auxin transport has been readily observed. Dynamic focal accumulation of PM proteins involved in the plant immune response has been documented at sites of pathogen infection (Frey and Robatzek, 2009). Proteomic analysis of the PM during diverse signaling events has led to a greater appreciation of plant PM complexity and plasticity (Simon-Plas et al., 2011; Urbanus and Ott, 2012). Label-based and label-free approaches can be employed for protein quantification at the level of proteins or peptides (Figure 1). The various PM proteome quantification strategies have been recently reviewed (Komatsu, 2008; Schulze and Usadel, 2010; Kline-Jonakin et al., 2011; Kota and Goshe, 2011).

Advances in quantitative fluorescence microscopy have also improved our understanding of PM protein movement within the membrane. Single molecule analysis of the PIP2;1 aquaporin has revealed disparate localizations and lateral mobilities in both non-stressed and salt-stressed cells indicating that this water channel is under complex regulation even under normal conditions (Li et al., 2011). Another recent study observed a range of different diffusion rates for a representative set of *Arabidopsis* PM proteins (Martinière et al., 2012). Furthermore, in contrast with other eukaryotes, the cytoskeleton and lipid microdomains had little effect on the mobilities of the proteins studied (Kusumi et al., 2012; Martinière et al., 2012). Interestingly, the plant cell wall was found to restrict the movement of proteins with domains projecting to the outer surface of the cell, suggesting that the plant cell wall can play a major role in the organization and mobilities of PM proteins (Martinière et al., 2012).

### Membrane microdomains

**Figure 2:**
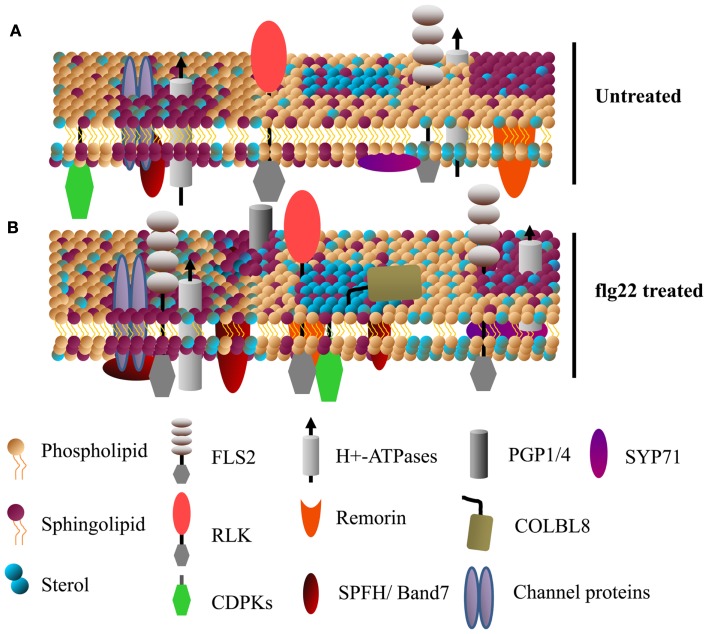
**Specific membrane proteins are enriched in detergent insoluble membranes upon activation of plant innate immunity**. A model showing the dynamics of detergent insoluble membrane (DIM) protein composition upon activation of plant innate immunity. **(A,B)** Show DIMs in untreated and flg22-treated *Arabidopsis* cell suspensions, respectively. Various receptor-like kinases (RLKs, including FLS2) and calcium dependent protein kinases (CDPKs) known to play a key role in plant defense singling are enriched in DIMs. Sytaxins (e.g., SYP71), transporter proteins (e.g., AHA1), ABC transporters (e.g., PGP1/4), the GPI-anchored protein COLBL8 (COBRA-like protein 8 precursor) are also some of the proteins rapidly associated with DIM after flg22 treatment (for details, see Keinath et al., 2010). Band 7 domain-containing proteins, known to be involved in membrane microdomain/vesicle formation, are among the proteins that rapidly associate with DIMs.

Dynamic, compositionally distinct regions exist within the PM that are implicated in the lateral compartmentalization of specialized signaling hubs and biological response pathways (Zappel and Panstruga, 2008; Simon-Plas et al., 2011; Urbanus and Ott, 2012). These membrane microdomains are enriched in sphingolipids and sterols relative to the rest of the membrane, which create a liquid-ordered phases distinct from the liquid-disordered membrane regions enriched in phospholipids (Figure 2). PM microdomains tend to contain characteristic proteins but are not static; their lipid and protein composition can be modulated during various signaling events (as recently reviewed in Mongrand et al., 2010; Simon-Plas et al., 2011; Cacas et al., 2012; Urbanus and Ott, 2012) (Figure 2). We should note that PM microdomain isolation using detergent insoluble membrane (DIM) preparations is prone to artifacts derived from isolation conditions and it is unlikely that DIMs preparations are equivalent to pre-existing microdomains *in vivo* (Tanner et al., 2011). Nevertheless, the utility of this technique in analyzing dynamic protein re-localization to DIMs during biological stimulus has been recently demonstrated (Minami et al., 2009; Keinath et al., 2010; Tanner et al., 2011). While plant PM microdomains are expected to have major roles in plant cell function and stress signaling, caution should be used when analyzing and interpreting proteins identified in DIM preparations.

### Abiotic stress and hormone signaling

The PM proteome mediates many cellular responses to environmental changes and hormone signaling. Numerous physiological adaptations of plants to cold stress occur at the PM (Kawamura and Uemura, 2003; Minami et al., 2009; Li et al., 2012). Substantial changes in the abundance of PM proteins were detected after cold or ABA treatment of *Arabidopsis* suspension cell cultures using label-free ion intensity quantification (Li et al., 2012). There was a significant overlap in protein regulation during cold stress and ABA treatment, suggesting that ABA signaling mediates cold tolerance (Li et al., 2012). Another study of DIM composition during cold acclimation found that the proteins and sterols present in DIMs are modulated when plants are exposed to freezing conditions, pointing to possible mechanisms of cell survival (Minami et al., 2009). Other studies have used ^15^N-metabolic labeling to study the effects of cadmium toxicity on PM protein regulation (Lanquar et al., 2007).

**Figure 3:**
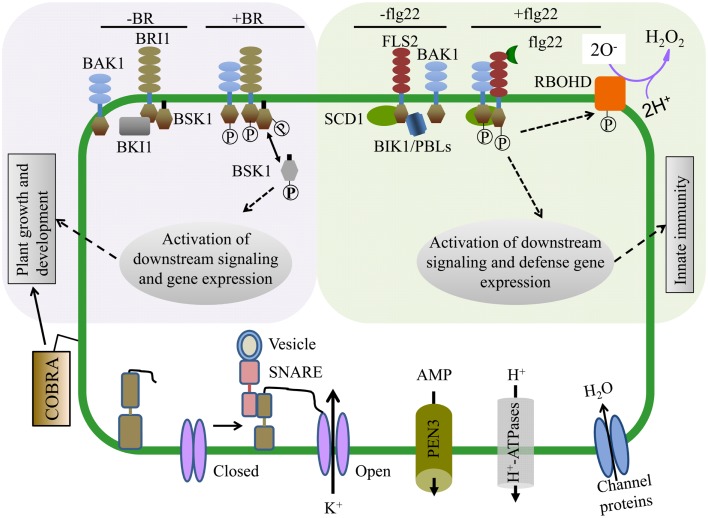
**Proteomics approaches have enabled mechanistic insight into hormone and pathogen perception as well as identified proteins required for cellular function**. (Upper Left) Brassinosteroids (BR) regulate plant growth and development and are perceived by the hormone receptor BRI1 (BR-insensitive 1). In the absence of BR, BKI1 inhibits BRI1 and its downstream signaling components. In the presence of BR, BRI1 associates with its co-receptor BAK1 and phosphorylates BSK1. BSK1 then disassociates from the BR receptor complex and plays key roles in phosphorylation dependent downstream signaling leading to transcriptional changes affecting plant growth and development. (Upper Right) The FLS2 (Flagellin Sensing 2) innate immune receptor recognizes a 22 amino acid epitope of the bacterial PAMP flagellin (flg22). In the presence of flg22, FLS2 interacts with its co-receptor BAK1 and multiple transphosphorylation events occur between the kinase domains of FLS2, BAK1, and BIK1/PBLs, leading to the activation of plant innate immunity and disease resistance. Within minutes of flg22 perception, the NADPH oxidase RBOHD is activated, potassium and calcium ion fluxes occur, and the apoplastis alkalinized. (Bottom) PM proteomics studies have also identified many proteins essential for both normal cellular homeostasis as well as signaling. The abundant GPI-anchored protein COBRA controls orientational cell expansion. Multiple integral PM proteins are ion transporters, ABC transporters (e.g., PEN3, transporting antimicrobial peptides), and water transporters. PM proteins can also dynamically interact with proteins from other compartments. For example, SNAREs like SYP121 (SNARE domain-containing syntaxin) play an important role in membrane fusion and shuttling of proteins between organelles. SYP121 mediates the association between itself, an R-SNARE and the PM potassium inward rectifying channel, leading to the opening of the potassium channel and transport across the membrane.

Brassinosteroids regulate a variety of plant growth and developmental processes. The PM-localized receptor kinase BRI1 directly binds BR at its extracellular domain and activates intracellular signaling (Tang et al., 2010; Clouse, 2011) (Figure 3). Proteomic examination of *Arabidopsis* BR responses at the PM identified proteins that change in abundance and/or phosphorylation status after BR treatment (Tang et al., 2008a,b, 2010; Wang et al., 2008; Karlova et al., 2009). Extensive phosphoproteomic analyses have identified specific regulatory sites in the somatic embryogenesis receptor-like kinase (SERK) family, whose members play diverse roles in mediating BR-signaling and immunity (Wang et al., 2005, 2008; Karlova et al., 2009; Tang et al., 2010). Phosphorylated forms of the BRI1-associated kinase BAK1 (SERK3) and the novel BR-signaling kinases BSK1 and BSK2 were detected by two-dimensional difference gel electrophoresis (2D-DIGE) shortly after BR treatment (Tang et al., 2008a,b). BRI1 phosphorylates BSK1 directly which releases it from the BRI1 PM complex and promotes its interaction with downstream cytoplasmic signaling components (Figure 3) (Tang et al., 2008b, 2010; Clouse, 2011). These studies highlight the advantages of using proteomic approaches to dissect complex signaling pathways and identify important, but genetically redundant signal mediators.

### Biotic stress

Many proteins that function in plant immune responses reside on or associate with the PM. Several studies have analyzed PM dynamics during pathogen perception and immune signaling. Protein phosphorylation has an extensive role in immune signaling and quantitative proteomics of phosphopeptides enriched from PM fractions isolated from tissue treated with pathogen-associated molecular patterns (PAMPs) has uncovered novel modes of protein regulation during immunity (Benschop et al., 2007; Nuhse et al., 2007). Plant defense response regulators RBOHD, SYP121, and PM H^+^-ATPase were differentially phosphorylated after PAMP application (Benschop et al., 2007; Nuhse et al., 2007). A subset of these phosphosites were also demonstrated to affect protein activity (Nuhse et al., 2007) (Figure 3). Thus, analysis of PTMs during pathogen recognition events has contributed to a mechanistic understanding of how immune regulators are activated.

Besides post-translational modifications, the local membrane environment of PM proteins is likely to affect enzyme activity, protein complex constituents, and signal transduction events. A study of PAMP-induced changes in ^15^N/^14^N-labeled *Arabidopsis* suspension cell cultures identified over 60 proteins that showed significant enrichment in DIM fractions within 15 min of flg22 (a 22 amino acid epitope of bacterial flagellin) treatment (Keinath et al., 2010). Among these, the flg22 receptor FLS2 abundance increased in DIMs, suggesting that rapid lateral compartmentalization of this receptor plays an important role in activation of downstream signaling. FLS2 undergoes endocytosis shortly after flg22 perception, and increased association with membrane microdomains could play a role in receptor endocytosis (Robatzek et al., 2006). In addition to FLS2, various other receptor kinases, PM H^+^-ATPases, Ca^2+^-ATPases, transporters, and characterized DIM-associated remorin and band 7 proteins showed enrichment in DIMs after flg22 treatment (Raffaele et al., 2009; Keinath et al., 2010; Qi et al., 2011) (Figure 2). The upregulation of known DIM markers in PM fractions after activation of plant immune receptors suggest that membrane microdomains have a significant role in plant disease resistance (Elmore et al., 2012).

While rapid protein re-localization to DIMs and post-translational modifications like phosphorylation can quickly modulate the plant immune response, the entire complement of PM proteins can change drastically over time during the execution of immunity. One study examined PM changes upon activation of the plant disease resistance protein RPS2, a signaling event that culminates in a form of programed cell death termed the hypersensitive response (HR) (Elmore et al., 2012). Relative protein quantification using spectral counting revealed that nearly 20% of the proteins identified in PM fractions significantly changed in abundance after RPS2 activation, revealing a striking alteration in PM composition during HR-associated immune responses (Elmore et al., 2012). Taken together, these studies highlight the dynamic nature of the plant PM during abiotic and biotic stress signaling and demonstrate the utility of PM proteomics approaches to study diverse biological processes.

Proteomic approaches have also been instrumental in identifying *Arabidopsis* immune-related PM protein complexes. Affinity purification-mass spectrometry (AP-MS) experiments have been instrumental in identifying interacting partners of the PAMP receptors FLS2 and EFR, the nucleotide binding-leucine repeat immune receptor RPS2, and the immune regulator RIN4 (Heese et al., 2007; Liu et al., 2009, 2011; Qi and Katagiri, 2009; Qi et al., 2011; Roux et al., 2011). It is likely that certain proteins exist in compositionally distinct complexes in different cell-types or even within the same cell. Future work using cell-type specific promoters driving expression of epitope-tagged proteins will facilitate the analysis of cell-type specific protein complexes. Thus, AP-MS experiments are an excellent tool for identifying potential interacting partners in PM protein complexes and provide a means to dissect how protein complexes are modulated under diverse signaling conditions.

## Conclusion and Future Prospects

Analysis of the *Arabidopsis* PM proteome over the last 15 years has uncovered many new insights into plant cell membrane structure and function. Recent studies have greatly advanced our understanding of PM microdomain behavior and receptor kinase-mediated signaling in *Arabidopsis* (Tang et al., 2010; Simon-Plas et al., 2011). Both top-down and bottom-up proteomics studies have been instrumental in the large-scale analysis of protein phosphorylation events during hormone and stress signaling, which otherwise would be impossible to study using alternative experimental approaches (Kline-Jonakin et al., 2011). Many other post-translational modifications control protein function, and we are only beginning to understand the intricacies of protein regulation. The development of proteomics approaches to study PTMs outside of phosphorylation will undoubtedly uncover additional layers of complexity in plant signaling networks.

Increases in the speed and sensitivity of the mass spectrometer instrument will soon facilitate virtually complete analysis of the PM proteome in a single experiment. Combining LC-MS/MS with cell biology approaches to survey PM responses to diverse stimuli will undoubtedly play an integral role in systems biology approaches for understanding complex cellular signaling events. Furthermore, combining quantitative proteomics with transcriptomics, metabolomics, and protein–protein interaction datasets will generate a wealth of testable models that will contribute to a holistic view of cell function.

## Conflict of Interest Statement

The authors declare that the research was conducted in the absence of any commercial or financial relationships that could be construed as a potential conflict of interest.
